# A model of extended technology acceptance for behavioral intention toward EVs with gender as a moderator

**DOI:** 10.3389/fpsyg.2022.1080414

**Published:** 2022-12-14

**Authors:** B. S. Zhang, Kashif Ali, Thavamaran Kanesan

**Affiliations:** ^1^Research Center of the Economic and Social Development of Henan East Provincial Joint, Shangqiu Normal University, Shangqiu, China; ^2^Department of Management Sciences, University Teknologi PETRONAS, Tronoh, Malaysia; ^3^Executive Office, Proofreading by A UK PhD, Cyberjaya, Malaysia

**Keywords:** behavioral intentions, technology acceptance model, perceived ease of use, perceived usefulness, gender, electric vehicles

## Abstract

Technology has contributed significantly to the adoption of EVs (EVs) in the era of industry 4. 0. However, consumer intentions for EVs have been elusive, and the pace of adoption has been confined. Therefore, this study aims to investigate the influence of external factors in promoting customer behavioral intention for EVs. The study also investigates the mediating role of perceived ease of use (PEU) and perceived usefulness (PU) between external factors and consumer intentions for EVs. It also examined the moderating role of gender on the study variables. A study approach based on the expanded version of the technology acceptance model (TAM) was utilized to analyse data from 203 customers in China. The model was tested using structural equation model (SEM) and multigroup analysis (MGA) techniques. The results indicated that two external factors have a positive relationship with TAM constructs. The results also indicate that PEU and PU have a serial mediating relationship between external factors and behavioral intention. Finally, the study revealed gender-related differences in TAM for EVs. The study's findings help managers to design successful strategies by knowing the external factors impacting customer EV intentions and gender differences. Finally, this is a ground-breaking research that applies TAM to the automobile sector. As a result, examining both new and current factors and evaluating them in a new setting adds to the body of the literature on the adoption of technology.

## Introduction

The development of the EVs industry is the result of the combination of technology, environment, and policy (Hossain et al., 2022). For the technology, the application of electronic information technology in the field of transportation plays an important role in the development of the smart city (Alonso et al., 2021). At the same time, energy-saving and low-pollution technologies will also contribute to the decarbonization of transportation and the development of low-carbon cities (Amini et al., 2017). For the policies, many countries, such as China, Malaysia, and South Korea, have issued policies to encourage the development of EVs (Adnan et al., 2018). In January 2022, the *National Development and Reform Commission of China* issued the *Implementation Plan for Promoting Green Consumption*, proposing to vigorously promote new-energy vehicles and promote consumption transformation. Such trends in technological innovation, environment, and policy commitments have put EVs in the fast lane, but EV adoption is low in most regions due to a variety of demand and supply-side market barriers (Mukherjee and Ryan, 2020). So, what factors influence consumers' adoption of EV technologies and their willingness to buy them? It becomes a research hotspot. The current research mainly includes technical and non-technical factors.

For the technology factor, it is undeniable that today's EVs have significant power reserves and incorporate many technological advancements that facilitate both consumer needs and the environment (Tran et al., 2020). Some of these studies also claimed that for EV customers, who value innovation, technological growth, and enhancement, technophilia had become a crucial selling point instead of other key environmental and hedonic motivational features (Chen and Li, 2020; Huang et al., 2021). Thiel et al. (2020) analyzed economic value and argued that EVs are significantly more technologically advanced than regular automobiles, increasing their worth to consumers. However, some studies have found that there are still obstacles to technology adoption (Krishna, 2021), and analysis amongst car consumers stated that the sentiment toward electric vehicle technology is predominantly negative (Jena and Singh, 2020).

For the non-technical factors, many studies focus on consumers' psychological factors such as value tendency, attitude, and emotion. First, the value concept based on solving social problems affects people's consumption intention. For example, people's concerns for the environment led to increased green behavior amongst people to adopt environmentally friendly products (Wang et al., 2021). With the increase in social problems, such as traffic congestion and accidents, the public's willingness toward using driverless vehicles tends to rely on their value predispositions, especially emotion, of paying attention to traffic safety (Ho et al., 2020). Payre et al. (2014) also argued that there is a strong positive correlation between people's attitude to use this new technology and their intention to use fully automated driving (FAD). Second, motivation based on consumption experience and enjoyment affects people's intentions. With the improvement of people's living quality, consumers pay more attention to consumption experience, which further promotes enterprises' experience-based innovation (Prahalad and Ramaswamy, 2003). Zhou et al. (2021) contended that hedonic motives play an important role in car purchase intention. However, Huang et al. (2021) found that the enjoyment derived from driving (hedonic motives) increased the adoption of EVs, which is not significant because a majority of the population in the study was from low-income households. Meanwhile, Rezvani et al. (2018) also found that hedonic motives are not preferred by those failing to believe in a strong social norm surrounding environment-friendly behavior.

Therefore, due to the rapid development of EV technology and consumption in China, whether new enjoyment of consumption experience, green innovative ideas, and attention to social issues can promote the adoption of technology is the theme of this study. Given the discussion earlier, the impact of hedonic motives such as perceived enjoyment (PE) on the willingness to adopt EVs is still relatively unknown. This provides an opportunity for further research. This study aims to investigate external factors such as perceived environment friendliness (PEF), perceived enjoyment (PE), and objective usability (OU) to determine consumers' intention to adopt EVs. An extended TAM model is provided to describe why individuals are inclined to purchase EVs. Three variables were used to assess consumers' behavioral intentions (PEF, PE, and OU), and the mediating role of perceived ease of use (PEU) and perceived usefulness (PU) was examined, as well as the moderating role of gender. The theoretical contribution of this study is to study its application in China's EVs market through an extended TAM model, which is helpful in enlightening the problems of experiment consumption and responsible consumption in the theory of consumer behavior. At the same time, it is also helpful for enterprises to strengthen the participation of stakeholders in the practice of responsible innovation and think from the perspective of customers.

## Theoretical framework and hypothesis development

### Technology acceptance model

Management psychology models in new technology adoption intentions include UTUAT, TRA, TAM, and DTPB (Alghazi et al., 2021). Amongst them, TAM described the relations between PU, PEU, and behavioral intention (Davis, 1989). By analyzing perceived behavioral control (PBC), attitudes, and subjective norms, TPB can help predict user intentions (Ajzen, 1991). The two main models, TAM and TPB, provide a sufficient basis for adoption intentions, although TAM is more effective in measuring user satisfaction (Yang and Zhou, 2011). In previous studies, scholars often used these models to assess people's responses to new technologies (Adamuthe and Mane, 2019).

In the study of the causal relationship among belief, attitude, intention, and behavior, TAM has effectively predicted individual behavior across a wide range of information technologies (Mustafa et al., 2021). TAM and its extended model have been used in recent studies to assess the public acceptability of new-energy vehicle technologies and the impact of various government subsidies on consumers' purchase intentions and to derive policy and regulatory effects in various research contexts (Hu et al., 2020; Yankun, 2020; Jaiswal et al., 2021). This is mainly because new technologies may enable EVs to have attractive features and functions (Yankun, 2020).

**Figure 1:**
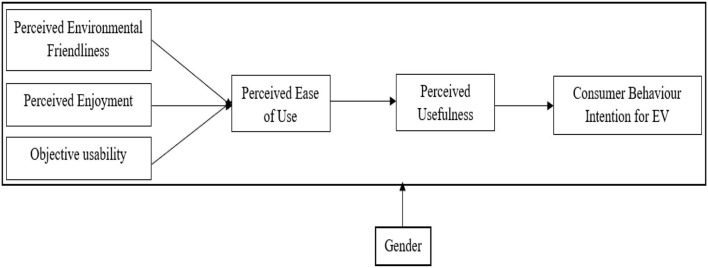
Research model.

Considering that PEF, PE, and OU may be the key external factors triggering consumer adoption of EV technology, the TAM model is extended in this study. Based on the theoretical and empirical research under the background of EV and TAM, the influence of these external factors on consumer PEU is studied. From an enterprise perspective, the outcome from PEF, PE, and OU could dramatically change the marketing strategies toward EV adoption. The relationships of PEU → PU and PU → CBIEV and the moderating effect of gender on the whole extended model relationship are also considered (see Figure 1).

### Hypothesis development

#### Relationship amongst constructs

In the prior literature, perceived environmental friendliness (PEF) describes products or services that have minimal to no impact on the environment (Reid et al., 2010). The past research studies affirmed that PEF was the enabling factor for future purchase intention and attitude. Joshi and Kronrod (2019) hypothesized that PEF has a positive association with purchase intention. The experimental study of Reid et al. (2010) argued that PEF help to promote sustainable vehicle design that ultimately affects consumer attitude. The empirical work of Hao et al. (2022) affirmed that the PEF of different packaging materials positively affects consumer buying behavior. Finally, Abbasi et al. (2021) argued that PEF knowledge has a positive relationship with electrical vehicle purchase intention. Based on the literature and theoretical support, the following hypothesis has been proposed.

**H1:** Perceived environmental friendliness has a positive relationship with perceived ease of use.

According to the TAM acceptance model, perceived enjoyment is related to intrinsic motivations, which promote the performance of an action for no reason other than the act of completing the action itself (Teo and Noyes, 2011). Perceived enjoyment (PE) is defined as the extent to which the activity of utilizing technology is perceived to be enjoyable in and of itself, independent of any expected performance benefits (Sun and Zhang, 2006). In past studies, perceived enjoyment (PE) has a strong association with perceived ease of use and other behavioral constructs. Holdack et al. (2020) argued that PE and PEU have positive linkages to promote digital technologies. Likewise, Sarosa (2019) hypothesized the association between PE and the intention to use iPod. Finally, Teo and Noyes (2011) affirmed the positive linkages between PE and PEU. The following hypothesis has been developed based on theoretical and literature support.

**H2:** Perceived enjoyment has a positive relationship with perceived ease of use.

In the TAM, objective usability (OU) is considered the external variable. It refers to compare different systems using objective measures of usability/systems characteristics (Venkatesh and Davis, 1996). They further argued that the higher the OU, the system is easier to use. Venkatesh (2000) argued that anchors and adjustments were the determinants of PEU, where PE and OU fall under the adjustments determinant. Bamigbola and Adetimirin (2020) hypothesized that adjustment factors (PE and OU) have a positive and significant relationship with PEU in Nigeria. Tao et al. (2020) affirmed that the OU has a significant effect on PEU in the health sector. Based on an earlier discussion, H3 has been proposed.

**H3:** Objective usability has a positive relationship with perceived ease of use.

Based on the theoretical framework of TAM, perceived ease of use (PEU) and usefulness (PU) are considered determinants of individual acceptance of a new technology and are much influenced by various external factors (Ma et al., 2017). In the past literature, PEU is associated with PU and leads to consumer purchase intention (Padilla-Meléndez et al., 2013). Tao et al. (2020) affirmed that PEU has a positive relationship with PU that further enhances customer behavioral intention. Therefore, the following hypotheses have been proposed.

**H4:** Perceived ease of use has a positive relationship with perceived usefulness.
**H5:** Perceived usefulness has a positive relationship with consumer behavior intention for EV.

#### Mediating role of perceived ease of use

In the technology adoption literature, perceived ease of use is considered the intervening construct between external factors and customer purchase intention (Marangunić and Granić, 2015). Moreover, the mediating role of PEU has been well-established in the literature. For instance, Chen and Aklikokou (2019) affirmed the mediating role of PEU as the determinant of e-government adoption in China. Mustapha and Obid (2015) hypothesized the mediating role of PEU on the relationship between online tax system usage and tax service quality. In addition, some scholars have studied the positive effect of external factors on PEU and PU and the positive effect of PEU on PU (Rezvani et al., 2022). Combined with these research documents, we put forward the following assumptions by discussing with relevant experts.

**H6a:** Perceived ease of use has a mediating role between perceived environmental friendliness and perceived usefulness.
**H6b:** Perceived ease of use has a mediating role between perceived enjoyment and perceived usefulness.
**H6c:** Perceived ease of use has a mediating role between objective usability and perceived usefulness.

#### Mediating role of perceived usefulness

In the technology acceptance literature, perceived usefulness (PU) is considered the intervening construct between external factors and customer purchase intention (Marangunić and Granić, 2015). Moreover, the mediating role of PU has been well-established in the literature. For instance, Chen and Aklikokou (2019) affirmed the mediating role of PU as the determinant of e-government adoption in China. Rezvani et al. (2022) argued that PU has a mediating role between external adoption factors and app use behavioral intention in Iran. Finally, Lui et al. (2021) examined the mediating effect of PU between external factors and the actual adoption of mobile payment in Malaysia. Thus, based on the discussion, the following hypotheses have been proposed.

**H7a:** PU has a mediating role between PEF → PEU and CBIEV.
**H7b:** PU has a mediating role between PE → PEU and CBIEV.
**H7c:** PU has a mediating role between OU → PEU and CBIEV.

#### Moderating role of gender

Marangunić and Granić (2015) argued that TAM has four major modifications, and gender as a contextual variable is one of them. Gefen and Straub (1997) argued that gender as moderator is considered an important contextual factor in TAM. The past literature argued that gender is considered an important factor in new technology adoption. For instance, Binyamin et al. (2020) examined the moderating role of gender on student acceptance of learning management systems in Saudi Arabia. Likewise, Na et al. (2021) examined the moderating effect of gender on the relationship between technology readiness and consumer intention of self-service in Korea. Merhi et al. (2021) examined the moderating role of gender on consumer intention to use mobile banking in Lebanese and British banks. Finally, Kim (2016) examined the moderating role of gender on the relationship technology acceptance model in hotel tablet apps. Therefore, the following hypothesis has been formulated.

**H8:** Gender has a moderating role in all exogenous and endogenous relationships.

## Methodology

### Sampling and data collection

The choice of sample population and method in the current study are based on the goals of the study. From January 2021 to April 2021, the survey was conducted in Shenzhen and Guangzhou with the sampled population consisting of consumers/drivers with knowledge of operating EVs. Both cities are situated in southern China and are the windows of China's economic opening up. From January 2020 to September 2020, Guangdong produced 83,800 new-energy vehicles, ranking second only to Shanghai, according to data from *the China Automobile Industry Statistical Yearbook 2021*. The number of charging piles is 86,000, second only to Beijing. In addition, according to the data of *the China City Statistical Yearbook in 2021*, by the end of 2020, the permanent residents of Guangzhou and Shenzhen were 18.74 million and 17.63 million, respectively, ranking the sixth and seventh in China. The GDP of Shenzhen and Guangzhou was 2,767 billion yuan and 2,501.9 billion yuan, respectively, ranking third and fourth in China. According to *the Guangdong Provincial Government's 14th Five-Year Plan for Energy Conservation and Emission Reduction*, by 2025, new-energy vehicle sales in the province will account for about 20% of total vehicle sales. In addition, BYD, China's well-known local brand of new-energy vehicles, has its corporate headquarters in Shenzhen. So, these two cities have certain representativeness. With the assistance of transportation and other relevant departments in the two cities, users were randomly selected for investigation. Before the investigation, every participant was informed in advance, and the information of the respondents was kept strictly confidential. Table 1 shows the descriptive statistics of study participants.

**Table 1:** Demographic characteristics of respondents.

**Variable**		**Frequency**	**Valid percent**
Gender	Female	92	45.32%
	Male	111	54.68%
Age	18–25	31	15.27%
	26–30	69	33.99%
	31–35	75	36.95%
	36 and Above	28	13.79%
Education	Diploma	41	20.20%
	Bachelors	103	50.74%
	Postgraduate	59	29.06%
Occupation	Unemployed	15	7.39%
	Employed	81	39.90%
	Self-Employed	107	52.71%
Income (RM)	4,851–5,880	3	1.47%
	5,881–7,100	48	23.64%
	7,100–8,700	63	31.03%
	8,701–10,970	89	43.85%

### Measures

In this research, the independent variables are perceived environmental friendliness (PEF), perceived enjoyment (PE), and objective usability (OU). All the study items are adapted from past studies. The items related to PEF are adapted from Wu et al. (2018) and Chen et al. (2015). The items related to PE are adapted from Holdack et al. (2020), and items related to OU are adapted from Bamigbola and Adetimirin (2020) and Tao et al. (2020). In this study, two mediator variables are used, perceived ease of use (PEU) and perceived usefulness (PU). The items related to PEU and PU are adapted from Sun and Zhang (2006), Bamigbola and Adetimirin (2020), and Tao et al. (2020). Finally, the behavioral intentions-related items are adapted from Tao et al. (2020) and Abbasi et al. (2021). All the items are measured on a 7-point Likert scale. In addition, the moderating variable (gender) is measured on a dichotomy scale, where “1” represents males and “2” represents females. The mean value and standard deviation of the questionnaire items are shown in Table 2. It indicates that all items have roughly equivalent means and standard deviations within a Likert scale (ratio of maximum standard deviation to minimum standard deviation of ~1.10:1).

**Table 2:** Descriptive statistics of items.

**Items**		***N***	**Mean**	**SD**	**MAX SD/MIN SD**
PEF	PEF1	203	3.97	2	1.10
	PEF2	203	4.08	2.08	
	PEF3	203	4.03	2.22	
	PEF4	203	4.07	2.07	
PE	PE1	203	4.56		1.07
	PE2	203	4.69	2.14	
	PE3	203	4.58	2.09	
	PE4	203	4.62	2.22	
	PE5	203	4.81	2.08	
	PE6	203	4.64	2.10	
OU	OU1	203	3.27		1.18
	OU2	203	3.36	1.91	
	OU3	203	3.25	2.14	
	OU4	203	3.59	2.13	
	OU5	203	3.41	2.21	
	OU6	203	3.35	2.17	
	OU7	203	3.40	2.25	
	OU8	203	4.06	2.22	
PEU	PEU1	203	4.17		1.07
	PEU2	203	4.32	2.13	
	PEU3	203	4.10	2.18	
	PEU4	203	4.25	2.24	
	PEU5	203	3.98	2.15	
PU	PU1	203	4.07		1.05
	PU2	203	4.12	2.09	
	PU3	203	3.95	2.18	
	PU4	203	4.03	2.09	
	PU5	203	4.09	2.17	
	PU6	203	4.10	2.11	
	PU7	203	4.02	2.07	
CBIEV	CBIEV1	203	4.13	2	1.05
	CBIEV2	203	4.04	2.16	
	CBIEV3	203	4.14	2.24	
	CBIEV4	203	4.03	2.15	

## Data analysis

### Descriptive statistics and common method bias

This section indicates the mean, standard deviation (S.D), skewness, and kurtosis of all study variables. Table 3 indicates the descriptive statistics in detail. The mean values of all study variables are above 3.5. Moreover, the skewness and kurtosis values fall within the threshold limit (±2) (George and Mallery, 2010). Moreover, to check the biases in the dataset, the common method bias (CMB) analysis is performed. Based on the recommendations of Podsakoff et al. (2003) both statistical and procedural remedies are adopted to minimize the CMB issue. Harman's single-factor test was performed from a statistical standpoint. The results show that a single factor accounted for <50% of the variance. In addition, Kock (2015) claimed that the gathered dataset is free from CMB if the value of VIF (variance inflation factor) during the full collinearity test is ≤ 3.3.

**Table 3:** Descriptive statistics.

**Variables**	**Mean**	**S.D**	**Skewness**	**Kurtosis**
PEF	4.022	1.882	0.007	−1.273
PE	4.599	1.801	−0.311	−1.065
OU	3.665	1.892	0.222	−1.220
PEU	4.071	2.027	−0.094	−1.380
PU	4.049	1.979	−0.042	−1.351
CBIEV	4.084	1.788	−0.079	−1.046

### Assessment of measurement model

#### Reliability and convergent validity

According to Hair et al. (2019), the PLS-SEM technique is divided into assessment and structural measurement models. The assessment of the measurement model consists of items loading, reliability, and convergent validity. Hair et al. (2019) recommended the items loading range from 0.50 to 0.85. For construct reliability, composite reliability (CR) and rho_A were adopted, and the threshold value of CR and rho_A is ≥0.70. For convergent validity, average variance extracted (AVE) is used, and the threshold value is ≥0.50. In addition, the variance inflated factor (VIF) is used, and the threshold value is ≤ 3.3 (Hair et al., 2019). Based on the reporting standards of past studies (Ali et al., 2022), this study presents the reliability and convergent validity results into three sub-sample (complete, male, and female). Table 4 shows the reliability (CR and rho_A), convergent validity (AVE), items loading, and VIF statistics. All the statistics are within the threshold limits.

**Table 4:** Reliability and convergent validity.

**Dimensions**	**Items**	**Complete**					**Male**					**Female**				
		**Loading**	**VIF**	**Reliability**		**AVE**	**Loading**	**VIF**	**Reliability**		**AVE**	**Loading**	**VIF**	**Reliability**		**AVE**
				**CR**	**rho_A**				**CR**	**rho_A**				**CR**	**rho_A**	
Perceived environmental friendliness	PEF1	0.872	2.369	0.926	0.895	0.757	0.879	2.538	0.930	0.901	0.768	0.876	2.240	0.920	0.899	0.742
	PEF2	0.864	2.340				0.862	2.274				0.855	2.509			
	PEF3	0.876	2.508				0.878	2.630				0.865	2.500			
	PEF4	0.868	2.504				0.887	2.926				0.848	2.257			
Perceived enjoyment	PE1	0.753	1.707	0.900	0.868	0.599	0.735	1.694	0.900	0.868	0.574	0.762	1.719	0.899	0.871	0.598
	PE2	0.780	1.892				0.804	2.081				0.777	1.872			
	PE3	0.768	1.874				0.795	2.035				0.723	1.746			
	PE4	0.797	1.954				0.779	1.964				0.807	2.137			
	PE5	0.773	1.839				0.769	1.902				0.775	1.812			
	PE6	0.773	1.814				0.762	1.946				0.795	1.836			
Objective usability	OU1	0.709	1.643	0.907	0.895	0.584	0.655	1.478	0.904	0.879	0.574	0.788	2.135	0.901	0.863	0.568
	OU2	0.715	1.654				0.730	1.708				0.738	1.689			
	OU3	0.793	1.882				0.766	1.872				0.624	2.003			
	OU5	0.730	1.760				0.750	1.795				0.827	1.830			
	OU6	0.757	1.810				0.744	1.757				0.766	1.911			
	OU7	0.794	2.015				0.790	2.068				0.745	2.034			
	OU8	0.842	2.336				0.854	2.582				0.770	2.176			
Perceived ease of use	PEU1	0.858	2.257	0.920	0.885	0.743	0.916	3.623	0.953	0.935	0.835	0.775	1.591	0.869	0.800	0.624
	PEU2	0.865	2.402				0.922	3.844				0.797	1.694			
	PEU3	0.844	2.091				0.915	3.500				0.747	1.450			
	PEU5	0.880	2.513				0.903	3.236				0.838	1.858			
Perceived usefulness	PU1	0.851	2.613	0.943	0.935	0.735	0.857	2.758	0.940	0.930	0.725	0.873	2.475	0.944	0.982	0.739
	PU2	0.868	2.824				0.878	3.017				0.849	2.675			
	PU3	0.841	2.649				0.820	2.418				0.860	3.059			
	PU4	0.863	2.858				0.857	2.700				0.842	3.133			
	PU5	0.852	2.765				0.837	2.557				0.852	3.221			
	PU6	0.869	2.700				0.858	2.584				0.881	2.882			
Consumer behavior intention for EV	CBIEV1	0.790	1.624	0.862	0.793	0.612	0.701	1.407	0.844	0.793	0.578	0.881	2.017	0.877	0.879	0.641
	CBIEV2	0.826	1.756				0.838	1.658				0.804	1.969			
	CBIEV3	0.810	1.674				0.829	1.668				0.785	1.719			
	CBIEV4	0.695	1.325				0.656	1.270				0.726	1.410			

#### Discriminant validity

The examination of the measurement model also includes discriminant validity in addition to reliability and convergent validity. It ensures that all variable is statistically distinct and captures unique phenomena (Hair et al., 2020). Different indicators are employed in discriminant validity, including Fornell–Larker, cross-loading, and the heterotrait–monotrait ratio (HTMT). According to Hair et al. (2017), Fornell–Larker and cross-loading misrepresent the prevalence of discriminant validity. As a result, Hair et al. (2019) suggested that HTMT should be employed for discriminant validity (Henseler et al., 2015). Hair et al. (2017) proposed that the HTMT threshold value for unique variables is less than 0.85 and for related constructs is <0.90. Table 5 shows the discriminant validity (HTMT) of three sub-samples, and all the values of HTMT are within the threshold limit (<0.90).

**Table 5:** Discriminant validity (HTMT).

	**Variables**	**PEF (1)**	**PE (2)**	**OU (3)**	**PEU (4)**	**PU (5)**	**CBIEV (6)**
Complete (203)	PEF (1)						
	PE (2)	0.391					
	OU (3)	0.743	0.399				
	PEU (4)	0.559	0.756	0.490			
	PU (5)	0.344	0.579	0.507	0.454		
	CBIEV (6)	0.494	0.378	0.353	0.476	0.250	
Male (111)	PEF (1)						
	PE (2)	0.639					
	OU (3)	0.834	0.791				
	PEU (4)	0.790	0.836	0.970			
	PU (5)	0.499	0.699	0.763	0.715		
	CBIEV (6)	0.500	0.392	0.570	0.604	0.340	
Female (92)	PEF (1)						
	PE (2)	0.111					
	OU (3)	0.624	0.151				
	PEU (4)	0.242	0.655	0.220			
	PU (5)	0.134	0.424	0.196	0.079		
	CBIEV (6)	0.485	0.351	0.199	0.335	0.150	

Previously, goodness-of-fit (GoF) was considered to have validity and to be possibly beneficial in a purely confirmatory method (Hair et al., 2019). However, the PLS-SEM approach requires an understanding of the causal and predictive relationships between constructs, as well as a description of theoretical components (Hair et al., 2019). As a result, no goodness-of-fit analysis is required to obtain the PLS-path model (Hair et al., 2019). As a result, no goodness-of-fit analysis is given in this research.

### Assessment of structural model and hypotheses testing (H1–H7)

To measure the structural model and hypotheses testing, bootstrapping technique was applied with 5,000 resamples (Henseler et al., 2015). As recommended by Hair et al. (2020), a hypothesis should be supported if the *p*-value is significant at 0.001–0.05, and the *t*-value is >1.645. Table 6 shows the structural model statistics. As reported in Table 6, the association PEF → PEU is significant (β = 0.261, *t*-value = 3.501, *p* < 0.05); thus, H1 has been accepted. Moreover, the relationship of PE → PEU is significant (β = 0.547, *t*-value = 9.436, *p* < 0.05); thus, H2 has been accepted. Unexpectedly, the relationship of OU → PEU is not significant (β = 0.078, *t*-value = 1.022, *p* = 0.307); thus, H3 has been rejected. Moreover, the relationship of PEU → PU is significant (β = 0.417, *t*-value = 6.657, *p* < 0.05); thus, H4 has been accepted. Finally, the relationship of PU → CBIEV is significant (β = 0.217, *t*-value = 3.385, *p* < 0.05); thus, H5 has been accepted.

**Table 6:** Structural model and effect size.

**Relationships**	**β-value**	***t*-value**	***p*-value**	***f* ^2^**	**Decision**
H1: PEF → PEU	0.261	3.501	0.000	0.080	Supported
H2: PE → PEU	0.547	9.436	0.000	0.540	Supported
H3: OU → PEU	0.078	1.022	0.307	0.007	Not supported
H4: PEU → PU	0.417	6.657	0.000	0.211	Supported
H5: PU → CBIEV	0.217	3.385	0.001	0.050	Supported
**Indirect effect**
H6a: PEF → PEU → PU	0.109	3.29	0.001		Supported
H6b: PE → PEU → PU	0.228	4.956	0.000		Supported
H6c: OU → PEU → PU	0.032	0.968	0.333		Not supported
H7a: PEF → PEU → PU → CBIEV	0.024	2.111	0.035		Supported
H7b: PE → PEU → PU → CBIEV	0.050	2.407	0.016		Supported
H7c: OU → PEU → PU → CBIEV	0.007	0.801	0.423		Not supported

**Figure 2:**
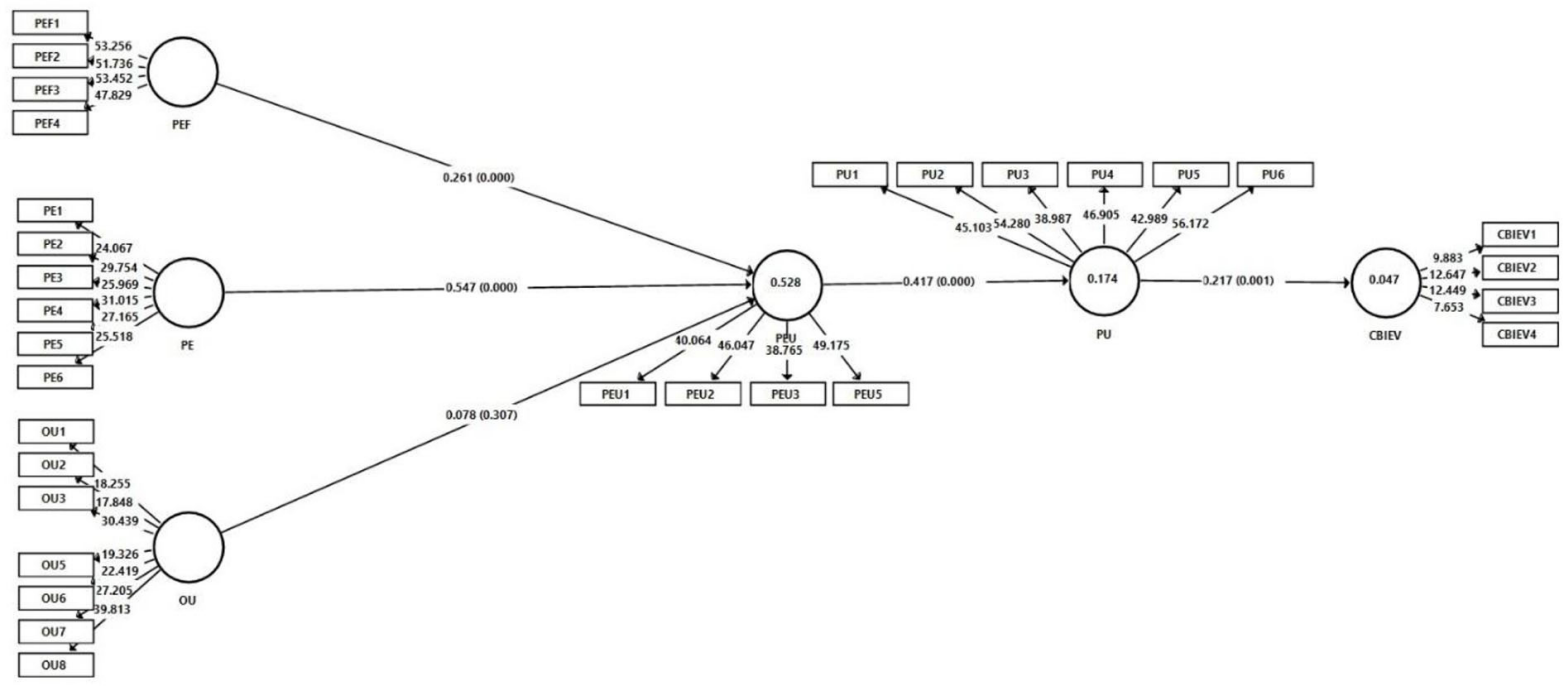
PLS-Path model.

In addition, Table 6 depicts the indirect/mediating role of PEU and PU between exogenous and endogenous variables. From Table 6, the relationship between PEF → PEU → PU (β = 0.109, *t*-value = 3.290, *p* < 0.05) and PE → PEU → PU (β = 0.228, *t*-value = 4.956, *p* < 0.05) is positive and significant. Thus, H6a and H6b support that PEU has a mediating role between PEF, PE, and PU. However, the relationship of OU → PEU → PU is not significant (β = 0.032, *t*-value = 0.968, *p* = 0.333). Therefore, H6c is rejected. Furthermore, the association of PEF → CBIEV (β = 0.024, *t*-value = 2.111, *p* < 0.05) and PE → CBIEV (β = 0.050, *t*-value = 2.407, *p* < 0.05) is mediated by PEU and PU. Thus, H7a and H7b have been accepted. Finally, the relationship of OU → CBIEV is not mediated by PEU and PU (β = 0.007, *t*-value = 0.801, *p* = 0.423). Therefore, H7c has not been supported. Figure 2 shows the PLS-path model.

### Multigroup analysis (H8)

The objective of a multigroup analysis (MGA) is to determine whether there is a substantial difference in path coefficients between two groups (male and female) (Ali et al., 2022). The analysis is divided into two phases (Henseler et al., 2016); measurement of invariance of composite models (MICOM) and PLS-MGA analysis. The MICOM approach is used to evaluate the measurement model's invariance in three phases. The MICOM analysis evaluates configurational invariance (step 1), compositional invariance (step 2), and the equality of composite mean values and variances (step 3). Partial configural invariance assessment and compositional invariance were established, as indicated in Table 7. However, in PEU, the equality of composite mean values and variances was not demonstrated. As a result, it is possible to conclude that only partial measurement invariance was demonstrated.

**Table 7:** Results of invariance measurement testing using permutation.

**Constructs**	**Step-1**	**Step-2**		**Partial measurement invariance established**	**Step-3**						**Full measurement invariance established**
	**Configural invariance (the same algorithms for both groups)**	**Compositional invariance**			**Equal mean assessment**			**Equal variance assessment**			
		**(Correlation = 1)**				
		**C = 1**	**5.00%**		**Differences**	**CI [2.5–97.5%]**	**Equal**	**Differences**	**CI [2.5–97.5%]**	**Equal**	
PEF (1)	Yes	0.999	0.997	Yes	0.202	[−0.282–0.271]	Yes	0.045	[−0.218-0.222]	Yes	Yes
PE (2)	Yes	0.999	0.997	Yes	0.234	[−0.277–0.278]	Yes	−0.084	[−0.267–0.249]	Yes	Yes
OU (3)	Yes	0.971	0.991	No	0.193	[−0.285–0.277]	Yes	0.018	[−0.241–0.236]	Yes	No
PEU (4)	Yes	1.000	0.999	Yes	0.521	[−0.273–0.279]	No	0.134	[−0.220–0.211]	Yes	No
PU (5)	Yes	0.996	0.997	No	0.237	[−0.280–0.301]	Yes	0.005	[−0.218–0.225]	Yes	No
CBIEV (6)	Yes	0.980	0.985	Yes	0.104	[−0.294–0.275]	Yes	−0.183	[−0.235–0.231]	Yes	Yes

PLS-MGA was done in the second phase utilizing Henseler's permutation technique (Henseler et al., 2016), as indicated in Table 8. The permutation method controls effectively for type I error and is more conservative than the parametric test. Furthermore, a lot of studies advocate using this method (Hair et al., 2017; Matthews, 2017).

**Table 8:** Permutation test path coefficient results.

**Relationship**	**β-value**	***p*-value**	**Decision**
PEF → PEU	−0.193	0.174	Not supported
PE → PEU	−0.214	0.047	Supported
OU → PEU	0.910	0.000	Supported
PEU → PU	0.612	0.000	Supported
PU → CBIEV	0.143	0.326	Not supported
PEF → PEU → PU	0.07	0.147	Not supported
PE → PEU → PU	0.154	0.016	Supported
OU → PEU → PU	0.415	0.001	Supported
PEU → PU → CBIEV	0.191	0.002	Supported
PEF → PEU → PU → CBIEV	0.024	0.129	Not supported
PE → PEU → PU → CBIEV	0.05	0.031	Supported
OU → PEU → PU → CBIEV	0.121	0.001	Supported

The results of Table 8 indicate that the direct relationship between PE → PEU and PEU → PU was significantly (*P* < 0.05) different between men and women. Although OU → PEU was significant, the direct relationship of OU → PEU was not significant according to the results of the H3 test earlier, so the moderating effect of gender here was invalid. Furthermore, the PLS-MGA indirect results indicate that PE → PEU → PU, PEU → PU → CBIEV, PE → PEU → PU → CBIEV, PE → PEU → PU → CBIEV were significantly (*P* < 0.05) different between men and women. Although moderating effects test of OU → PEU → PU, OU → PEU → PU → CBIEV are significant here, the above indirect relationship test of H6c is not significant, so the gender regulation effect here is also invalid.

## Conclusion

The article has three conclusions. First, PEF and PE have significant influences on PEU, but the EU has no significant influences on PEU. Second, in the mediation effects, due to the positive relationships of PEU → PU and PU → CBIEV, it is further found that PEU and PU play a mediating role between external TAM factors (PEF and PE) and CBIEV. Finally, in the moderating effects, it is found that gender has partial moderating roles in the whole extended TAM model.

## Discussion and implications

### Discussion of findings

The purpose of this study is to examine the role of various determinants to promote consumer behavioral intention for electrical vehicles with the moderating role of gender. Based on the technology acceptance model (TAM), a research framework has been drawn. To achieve the research objectives, eight hypotheses (H1–H8) have been formulated.

The first research hypothesis affirmed that PEF has a positive and significant relationship with PEU. The PLS-SEM analysis confirmed the association. These findings are consistent with past studies. Reid et al. (2010) argued that PEF was the most preferred dimension for vehicle design which elicit customer purchase behavior. Likewise, Joshi and Kronrod (2019) argued that PEF is considered essential for green product purchase intention. Thus, H1 was accepted. The second hypothesis affirmed that PE has a positive relationship with PEU. The empirical findings confirmed the relationship. These outcomes are in line with past literature. The empirical study of Teo and Noyes (2011) concluded that PE has a positive relationship with PEU. Moreover, past studies confirm that PE has a positive effect on TAM constructs (Sarosa, 2019; Holdack et al., 2020). Thus, H2 was accepted. The third hypothesis affirmed that OU has a positive relationship with PEU. Unexpectedly, the empirical findings depicted that the relationship between OU and PEU is not significant. These findings are supported by the empirical work of Tao et al. (2020). They found that OU has no significant relationship with PEU. Tao et al. (2020) argued that customer purchase intentions are not based on OU as their perceptions are based on subject performance which may be influenced by social and subject bias. Thus, H3 has been rejected. That is to say, perceptions based on the green concept and personal emotion are more likely to affect consumers' purchase intention than the objective use value of the product itself. What factors influence this perception and emotion? According to the AlixPartners 2021 EV consumer sentiment survey, 23% of those who have become interested in EVs in 2020 (including 36% in the United States) say friends and family are their biggest purchase influencers. Zhang et al. (2022) argue that the nudge policy of information propaganda promotion has a considerable promotion effect on EV adoption, but the consumers' over-communication will have negative effects on the adoption of EVs. So, how propaganda campaigns can influence people's willingness and behavior is very important.

The fourth and fifth hypotheses affirmed that the relationship between PEU → PU and PU → CBIEV have positive and significant. The statistical findings confirmed the relationships. These findings are consistent with past studies. Binyamin et al. (2020) affirmed that PEU has a positive relationship with PU that promotes behavioral intentions. Likewise, Ma et al. (2017) affirmed that PEU has a positive relationship with PU that further enhances customer purchase intention. Furthermore, Huang et al. (2021) argued that PEU has a positive relationship with PU and consumer intentions to adopt EVs in China. Therefore, H4 and H5 have been accepted.

The sixth and seventh hypotheses affirmed the partial mediating role of PEU and PU between TAM external factors and consumer behavioral intention of EV. The findings depicted that PEU and PU have a mediating role between TAM external factors (PEF and PE) and CBIEV but insignificant between OU and CBIEV. The study findings are consistent with past literature. Chen and Aklikokou (2019) affirmed that PEU and PU have a mediating role between technology factors and behavioral intention to use. Likewise, Dokhanian et al. (2022) concluded that PEU and PU have a mediating role between new technology external factors and behavioral intention. Therefore, H6a, H6b, H7a, and H7b have been accepted, and H6c and H7C have been rejected.

Finally, the eighth hypothesis affirmed that gender has a moderating effect on TAM dimensions relationship. The empirical findings indicate that gender has not a complete moderating effect on exogenous and endogenous relationships, but it has a partial moderating effect. Because the direct relationship between OU → PEU is not significant, the indirect relationship between OU → PEU → PU and OU → PEU → PU → CBIEV is not significant, so the moderating effect of gender is not considered. Therefore, mediating effects are mainly in PE → PEU, PEU → PU, PE → PEU → PU, PEU → PU → CBIEV, and PE → PEU → PU → CBIEV. Table 8 shows the results in detail. The absence of gender differences is understandable because of the neutral nature of environmental friendliness. But there are significant differences between women and men in the progressive relationship based on subjective emotion (PE). Therefore, H8 has been accepted. As to why PE is different for different genders. Harris and Jenkins (2006) argue that women's greater perceived likelihood of negative outcomes and lesser expectation of enjoyment partially mediated their lower propensity toward risky choices in gambling, recreation, and health domains. However, because men score lower on all the sources of shopping enjoyment than women, there are significant gender differences on all the sources of shopping enjoyment (Kotzé et al., 2012).

### Contributions and implications

The purpose of this study is to examine the direct effect of TAM constructs on customer behavioral intentions for EV and the indirect effect through perceived ease of use and usefulness. The study findings have a number of contributions. First, this study contributes to the literature on an extended version of the Technology Acceptance Model in EVs. Unlike the previous studies, this study highlights the role of external (PEF) and adjustment (PE and OU) variables in promoting EV purchase intention. Second, the suggested model incorporates components that have been examined in other contexts; this study takes it a step further by examining the indirect impact with perceived ease of use and perceived usefulness as mediating variables. Few researches on EV adoption examined the mediation effect of PEU and PU through the lens of the TAM framework. As a result, this study demonstrated the significance of PEU and PU as not only the key predictors of consumer behavioral intentions for EV but also as mediating variables. Finally, by including gender as moderating variable, this study adds insight into the scholarly discussion around their roles in the adoption of electric cars. The MGA results in this study demonstrated that PE and OU differed between male and female respondents. In summary, this study finds that PEF and PE are the primary predictors of EV behavioral intentions, PEU and PU are the serial mediators, and gender is a prominent moderator.

As this study is novel in addressing the adoption of EV vehicles, this study provides some practical implications. First, given the important and positive aspects of PEF, PE, and impairing their usefulness (PEU and PU) may be an effective way to rise customer EV purchase intention. This implication is consistent with past studies (Huang et al., 2021). EV producers might share technology information regarding EVs, such as how EVs are integrated with intelligent networking technologies, EV technological application scenarios, and EV technological performance, all of which would increase customer interest. Secondly, the MGA findings vary significantly between men and women. This suggests that women are more responsive to EVs' enjoyment and ease of use than men. As a result, the importance of gender in the choice to adopt EVs should not be overlooked, especially when purchasing a family automobile. Manufacturers may find it beneficial to spend more resources on demonstrating the enjoyment and ease of use of EVs to attract more female consumers. Thirdly, the support of H1, H4, and H5 indicates that meeting social expectations and environmental friendliness in the process of innovation is the current requirement of customer-centered responsible innovation (Zahinos et al., 2013). The inclusion dimension of responsible innovation (Stilgoe et al., 2013) promotes firms to pay more attention to the participation of stakeholders in the innovation process.

### Limitations and future research directions

This study has certain limitations that will serve as future guide studies. The current research utilized a sample of consumers from China; hence the generalizability of the study's findings is restricted. This study can be replicated in various industrial contexts with more varied consumer categories in future research. Future research can potentially enhance this study's model by changing relevant parameters based on the cultural background of the study participants or the type of vehicles utilized. The shortcoming of TAM is that it uses consumers' intentions for EVs as a dependent variable rather than actual EV usage behavior. Despite earlier research indicating that an individual's behavioral intention is a good predictor of his or her actual behavior (Davis, 1989). In the future study, the relationship between the proposed model and actual behavioral intention may be investigated. Moreover, in this research, only gender was considered as a moderator under the TAM framework. In future study, the role of other contextual (age and income level), cognitive (spatial and processing), and emotional (anxiety and fear of failure) factors might also contribute to the explanation of TAM.

## Data availability statement

The raw data supporting the conclusions of this article will be made available by the authors, without undue reservation.

## Author contributions

Concept, analysis, original draft, and funding: BZ. Proofreading, concept, analysis, and review: TK. Writing, analysis, concept, and review: KA. All authors contributed to the article and approved the submitted version.

## References

[B1] Abbasi H. A. Johl S. K. Shaari Z. B. H. Moughal W. Mazhar M. Musarat M. A.. (2021). Consumer motivation by using unified theory of acceptance and use of technology towards EVs. Sustainability. 13, 12177. 10.3390/su132112177

[B2] Adamuthe A. C. Mane S. U. (2019). Analyzing the adoption of recent IT technologies in undergraduate engineering project course. J. Eng. Educ. Transform. 34. 10.16920/jeet/2021/v34i0/157105

[B3] Adnan N. Md Nordin S Hadi Amini M. Langove N. (2018). What make consumer sign up to PHEVs? Predicting Malaysian consumer behavior in adoption of PHEVs. Transportation Research Part A: Policy and Practice. 113, 259–278. 10.1016/j.tra.2018.04.007

[B4] Ajzen I. (1991). The theory of planned behavior. Organ. Behav. Hum. Decis. Process. 50, 179–211. 10.1016/0749-5978(91)90020-T

[B5] Alghazi S. S. Kamsin A. Almaiah M. A. Wong S. Y. Shuib L. (2021). For sustainable application of mobile learning: an extended utaut model to examine the effect of technical factors on the usage of mobile devices as a learning tool. Sustainability. 13, 1856. 10.3390/su13041856

[B6] Ali K. Johl S. K. Muneer A. Alwadain A. Ali R. F. (2022). Soft and hard total quality management practices promote industry 4.0 readiness: a SEM-neural network approach. Sustainability 14, 11917. 10.3390/su141911917

[B7] Alonso F. Faus M. Esteban C. Useche S. A. (2021). Is there a predisposition towards the use of new technologies within the traffic field of emerging countries? The case of the dominican republic. Electronics. 10, 1208. 10.3390/electronics10101208

[B8] Amini M. H. Moghaddam M. P. Karabasoglu O. (2017). Simultaneous allocation of electric vehicles' parking lots and distributed renewable resources in smart power distribution networks. Sustain. Cities Soc. 28, 332–342. 10.1016/j.scs.2016.10.006

[B9] Bamigbola A. A. Adetimirin A. E. (2020). Assessing determinants of perceived ease of use of institutional repositories by lecturers in Nigerian universities. Int. Inf. Libr. Rev. 52, 95–107. 10.1080/10572317.2019.1662261

[B10] Binyamin S. S. Rutter M. J. Smith S. (2020). The moderating effect of gender and age on the students' acceptance of learning management systems in Saudi higher education. Knowl. Manag. E-Learn. 12, 30–62. 10.34105/j.kmel.2020.12.003

[B11] Chen X. Li Z. (2020). Research on the behavior of college students' online tourism booking based on TAM. J. Serv. Manag. 13, 28. 10.4236/jssm.2020.131003

[B12] Chen L. Aklikokou A. K. (2019). Determinants of e-government adoption: testing the mediating effects of perceived usefulness and perceived ease of use. Int. J. Public Adm. 43, 850–865. 10.1080/01900692.2019.1660989

[B13] Chen Y.-S. Lin C.-Y. Weng C.-S. (2015). The influence of environmental friendliness on green trust: the mediation effects of green satisfaction and green perceived quality. Sustainability 7, 10135–10152. 10.3390/su70810135

[B14] Davis F. D. (1989). Perceived usefulness, perceived ease of use, and user acceptance of information technology. MIS Quarterly. 13, 319–340. 10.2307/249008

[B15] Dokhanian S. Roustapisheh N. Heidari S. Rezvani S. (2022). The effectiveness of system quality, habit, and effort expectation on library application use intention: the mediating role of perceived usefulness, perceived ease of use, and user satisfaction. Int. J. Bus. Inf. Syst. 1, 1–18. 10.1504/IJBIS.2022.10049515

[B16] Gefen D. Straub D. W. (1997). Gender differences in the perception and use of e-mail: An extension to the technology acceptance model. MIS Quart. 389–400. Available online at: http://www.jstor.org/stable/249720

[B17] George D. Mallery P. (2010). SPSS for Windows Step by Step: A Simple Guide and Reference 17.0 Update (10th ed.). Pearson.

[B18] Hair J. F Jr Howard M. C. Nitzl C. (2020). Assessing measurement model quality in PLS-SEM using confirmatory composite analysis. J. Bus. Res. 109, 101–110. 10.1016/j.jbusres.2019.11.069

[B19] Hair J. Hollingsworth C. L. Randolph A. B. Chong A. Y. L. (2017). An updated and expanded assessment of PLS-SEM in information systems research. Ind. Manag. Data Syst. 117, 442–458. 10.1108/IMDS-04-2016-0130

[B20] Hair J. F. Risher J. J. Sarstedt M. Ringle C. M. (2019). When to use and how to report the results of PLS-SEM. *Eur. Bus. Rev*. 31, 2–24. 10.1108/EBR-11-2018-0203

[B21] Hao J. Cheng Z. de Regt A. (2022). “Assessing the perceived environmental friendliness of different packaging materials: an abstract,” in From Micro to Macro: Dealing with Uncertainties in the Global Marketplace. AMSAC 2020. Developments in Marketing Science: Proceedings of the Academy of Marketing Science, eds F. Pantoja and S. Wu (Cham: Springer). 10.1007/978-3-030-89883-0_16

[B22] Harris C. R. Jenkins M. (2006). Gender differences in risk assessment: why do women take fewer risks than men? Judgm. Decis. Mak. 1, 48–63. 10.1037/e511092014-21211398025

[B23] Henseler J. Ringle C. M. Sarstedt M. (2015). A new criterion for assessing discriminant validity in variance-based structural equation modeling. J. Acad. Mark. Sci. 43, 115–135. 10.1007/s11747-014-0403-8

[B24] Henseler J. Ringle C. M. Sarstedt M. (2016). Testing measurement invariance of composites using partial least squares. Int. Mark. Rev. 33, 405–431. 10.1108/IMR-09-2014-0304

[B25] Ho S. S. Leow V. J. X. Leung Y. W. (2020). Driving without the brain? Effects of value predispositions, media attention, and science knowledge on public willingness to use driverless cars in Singapore. Transportation Research Part F: Traffic Psychology and Behaviour. 71, 49–61. 10.1016/j.trf.2020.03.019

[B26] Holdack E. Lurie-Stoyanov K. Fromme H. F. (2020). The role of perceived enjoyment and perceived informativeness in assessing the acceptance of AR wearables. J. Retail. Consum. Serv. 102259. 10.1016/j.jretconser.2020.102259

[B27] Hossain M. S. Kumar L. Islam M. M.. (2022). A comprehensive review on the integration of electric vehicles for sustainable development. J. Adv. Transp. 10.1155/2022/3868388

[B28] Hu Y. Wang Z. Li X. (2020). Impact of policies on electric vehicle diffusion: An evolutionary game of small world network analysis. J. Clean. Prod. 265, 121703. 10.1016/j.jclepro.2020.121703

[B29] Huang X. J. Lin Y. Lim M. Tseng M. L. Zhou F. L. (2021). The influence of knowledge management on adoption intention of EVs: perspective on technological knowledge. Ind. Manag. Data Syst. 121, 1481–1495. 10.1108/IMDS-07-2020-0411

[B30] Jaiswal D. Kant R. Singh P. K. Yadav R. (2021). Investigating the role of electric vehicle knowledge in consumer adoption: evidence from an emerging market. Benchmarking: An International Journal. 10.1108/BIJ-11-2020-0579

[B31] Jena P. Singh R. K. (2020). “Mix energy source unified loop based dual active bridge for electric vehicle.” in IECON 2020 The 46th Annual Conference of the IEEE Industrial Electronics Society. IEEE, 2584–2589.

[B32] Joshi P. Kronrod A. (2019). Sounds of green: how brand name sounds metaphorically convey environmental friendliness. J. Advert. 49, 61–77. 10.1080/00913367.2019.1696720

[B33] Kim J. S. (2016). An extended technology acceptance model in behavioral intention toward hotel tablet apps with moderating effects of gender and age. Int. J. Contemp. Hosp. 28, 1535–1553. 10.1108/IJCHM-06-2015-0289

[B34] Kock N. (2015). Common method bias in PLS-SEM: A full collinearity assessment approach. International Journal of e-Collaboration (ijec) 11, 1–10.

[B35] Kotzé T. North E. Stols M. Venter L. (2012). Gender differences in sources of shopping enjoyment. Int. J. Consum. Stud. 36, 416–424. 10.1111/j.1470-6431.2011.01060.x

[B36] Krishna G. (2021). Understanding and identifying barriers to electric vehicle adoption through thematic analysis. Transp. Res. Interdiscip. Perspect. 10, 100364. 10.1016/j.trip.2021.100364

[B37] Lui T. K. Zainuldin M. H. Yii K. J. Lau L. S. Go Y. H. (2021). Consumer adoption of alipay in Malaysia: the mediation effect of perceived ease of use and perceived usefulness. Pertanika J. Soc. Sci. 29. 10.47836/pjssh.29.1.22

[B38] Ma Y. J. Gam H. J. Banning J. (2017). Perceived ease of use and usefulness of sustainability labels on apparel products: application of the technology acceptance model. Fash. Text. 4, 1–20. 10.1186/s40691-017-0093-1

[B39] Marangunić N. Granić A. (2015). Technology acceptance model: a literature review from 1986 to 2013. Univers. Access. Inf. Soc. 14, 81–95. 10.1007/s10209-014-0348-1

[B40] Matthews L. (2017). “Applying multigroup analysis in PLS-SEM: A step-by-step process,” in Partial Least Squares Path Modeling (Cham: Springer), 219–243.

[B41] Merhi M. Hone K. Tarhini A. Ameen N. (2021). An empirical examination of the moderating role of age and gender in consumer mobile banking use: a cross-national, quantitative study. J. Enterp. Inf. Manag. 34, 1144–1168. 10.1108/JEIM-03-2020-0092

[B42] Mukherjee S. C. Ryan L. (2020). Factors influencing early battery electric vehicle adoption in Ireland. Renewable Sustainable Energy Rev. 118, 109504. 10.1016/j.rser.2019.109504

[B43] Mustafa M. H. Ahmad M. B. Shaari Z. H. Jannat T. (2021). Integration of TAM, TPB, and TSR in understanding library user behavioral utilization intention of physical vs. E-book format. J. Acad. Librariansh. 47, 102399. 10.1016/j.acalib.2021.102399

[B44] Mustapha B. Obid S. N. B. S. (2015). Tax service quality: the mediating effect of perceived ease of use of the online tax system. Contemporary Issues in Management and Social Science Research. 172, 2–9. 10.1016/j.sbspro.2015.01.328

[B45] Na T.-K. Lee S.-H. Yang J.-Y. (2021). Moderating effect of gender on the relationship between technology readiness index and consumers' continuous use intention of self-service restaurant kiosks. Information 12, 280. 10.3390/info12070280

[B46] Padilla-Meléndez A. del Aguila-Obra A. R. Garrido-Moreno A. (2013). Perceived playfulness, gender differences and technology acceptance model in a blended learning scenario. Comput. Educ. 63, 306–317. 10.1016/j.compedu.2012.12.014

[B47] Payre W. Cestac J. Delhomme P. (2014). Intention to use a fully automated car: Attitudes and a priori acceptability. Transportation research part F: traffic psychology and behaviour. 27, 252–263. 10.1016/j.trf.2014.04.009

[B48] Podsakoff P. M. MacKenzie S. B. Lee J. Y. Podsakoff N. P. (2003). Common method biases in behavioral research: a critical review of the literature and recommended remedies. Am. J. Appl. Psychol. 88, 879–903. 10.1037/0021-9010.88.5.879 14516251

[B49] Prahalad C. K. Ramaswamy V. (2003). The new frontier of experience innovation. MIT Sloan Manag. Rev. 44, 12. Available online at: https://sloanreview.mit.edu/article/the-new-frontier-of-experience-innovation/

[B50] Reid T. N. Gonzalez R. D. Papalambros P. Y. (2010). Quantification of perceived environmental friendliness for vehicle silhouette design. J. Mech. Des. 132, 101010. 10.1115/1.4002290

[B51] Rezvani S. Heidari S. Roustapisheh N. Dokhanian S. (2022). The effectiveness of system quality, habit, and effort expectation on library application use intention: the mediating role of perceived usefulness, perceived ease of use, and user satisfaction. Int. J. Bus. Inf. Syst. 1, 1–18.

[B52] Rezvani Z. Jansson J. Bengtsson M. (2018). Consumer motivations for sustainable consumption: the interaction of gain, normative and hedonic motivations on electric vehicle adoption. Bus. Strategy Environ. 27, 1272–1283. 10.1002/bse.2074

[B53] Sarosa S. (2019). The role of brand reputation and perceived enjoyment in accepting compulsory device's usage: Extending UTAUT. Procedia Comput. Sci. 161, 115–122. 10.1016/j.procs.2019.11.106

[B54] Stilgoe J. Owen R. Macnaghten P. (2013). Developing a framework for responsible innovation. Res. Policy. 42, 1568–1580. 10.1016/j.respol.2013.05.008

[B55] Sun H. Zhang P. (2006). Causal relationships between perceived enjoyment and perceived ease of use: An alternative approach. J. Assoc. Inf. Syst. 7, 24. Available online at: https://aisel.aisnet.org/jais/vol7/iss1/24

[B56] Tao D. Shao F. Wang H. Yan M. Qu X. (2020). Integrating usability and social cognitive theories with the technology acceptance model to understand young users' acceptance of a health information portal. J. Health Inform. 26, 1347–1362. 10.1177/1460458219879337 31603378

[B57] Teo T. Noyes J. (2011). An assessment of the influence of perceived enjoyment and attitude on the intention to use technology among pre-service teachers: A structural equation modeling approach. Comput Educ. 57, 1645–1653. 10.1016/j.compedu.2011.03.002

[B58] Thiel C. Tsakalidis A. Jäger-Waldau A. (2020). Will EVs be killed (again) or are they the next mobility killer app? Energies. 13, 1828. 10.3390/en13071828

[B59] Tran M.-K. Sherman S. Samadani E. Vrolyk R. Wong D. Lowery M.. (2020). Environmental and economic benefits of a battery electric vehicle powertrain with a zinc–air range extender in the transition to EVs. Vehicles. 2, 398–412. 10.3390/vehicles2030021

[B60] Venkatesh V. (2000). Determinants of perceived ease of use: Integrating control, intrinsic motivation, and emotion into the technology acceptance model. Inf. Syst. Res. 11, 342–365. 10.1287/isre.11.4.342.11872

[B61] Venkatesh V. Davis F. D. (1996). A model of the antecedents of perceived ease of use: Development and test. Decis. Sci. 27, 451–481. 10.1111/j.1540-5915.1996.tb01822.x

[B62] Wang H. Y. Wang X. L. Sarkar A. Zhang F. H. (2021). How capital endowment and ecological cognition affect environment-friendly technology adoption: a case of apple farmers of Shandong Province, China. Int. J. Environ. Res. Public Health. 18, 7571. 10.3390/ijerph18147571 34300022 PMC8305192

[B63] Wu H.-C. Cheng C.-C. Chen Y.-C. Hong W. (2018). Towards green experiential loyalty: Driving from experiential quality, green relationship quality, environmental friendliness, green support and green desire. *Int. J. Contemp. Hosp. Manag*. 30, 1374–1397. 10.1108/IJCHM-10-2016-0596

[B64] Yang H. C. Zhou L. (2011). Extending TPB and TAM to mobile viral marketing: An exploratory study on American young consumers' mobile viral marketing attitude, intent and behavior. J. Target. Meas. Anal. Mark. 19, 85–98. 10.1057/jt.2011.11

[B65] Yankun S. (2020). “An empirical study on the influencing factors of consumers' willingness to use pure electric vehicle based on TAM Model.” in 2020 16th Dahe Fortune China Forum and Chinese High-educational Management Annual Academic Conference (DFHMC). 10.1109/DFHMC52214.2020.00063

[B66] Zahinos A. Singh R. González-Benítez M. (2013). “Moving toward responsible innovation approach in the automotive industry: The SEAT Case 9.” in Conference responsible innovation, The Hague, The Netherlands.

[B67] Zhang Q. Liu J. Yang K. Liu B. Wang G. (2022). Market adoption simulation of electric vehicle based on social network model considering nudge policies. Energy. 259, 124984. 10.1016/j.energy.2022.124984

[B68] Zhou M. Long P. Kong N. Zhao L. Jia F. Campy K. S. (2021). Characterizing the motivational mechanism behind taxi driver's adoption of EVs for living: Insights from China. Transportation Research Part A: Policy and Practice. 144, 134–152. 10.1016/j.trb.2021.01.002

